# Present but Ignored: Physical Condition and Health-Related Quality of Life in College-Aged Females with Generalized Joint Hypermobility

**DOI:** 10.3390/healthcare12111065

**Published:** 2024-05-23

**Authors:** Ernesta Aukštuolytė-Bačienė, Algė Daunoravičienė, Vilma Tamulionytė, Kristina Berškienė, Jurgita Narbutaitė, Selen Razon, Agnė Slapšinskaitė-Dackevičienė

**Affiliations:** 1 Department of Sports Medicine, Faculty of Nursing, Lithuanian University of Health Sciences, 47181 Kaunas, Lithuania; ernesta.aukstuolyte@lsmu.lt (E.A.-B.); alge.daunoraviciene@lsmu.lt (A.D.); vilma.tamulionyte@lsmu.lt (V.T.); kristina.berskiene@lsmu.lt (K.B.); jurganarbutaite@gmail.com (J.N.); 2 Department of Kinesiology, College of Health Sciences, West Chester University of Pennsylvania, West Chester, PA 19383, USA; srazon@wcupa.edu; 3 Health Research Institute, Lithuanian University of Health Sciences, 47181 Kaunas, Lithuania

**Keywords:** balance, explosive strength, foot posture, general joint hypermobility, handgrip strength, health related quality of life, trunk muscle static strength endurance

## Abstract

Background: Generalized joint hypermobility (GJH) is prevalent among young adults, necessitating effective monitoring of musculoskeletal health, particularly among college-aged females. This study aimed to identify physical fitness and health-related quality of life (HR-QoL) characteristics associated with GJH. Methods: A total of 67 participants were assessed: 26 with GJH (mean age 20.06, SD 1.2 years), and 41 without (mean age 20.15, SD 2.2 years). Assessments included hypermobility, anthropometric data, foot posture, balance, flexibility, strength, and HR-QoL. We used the Mann–Whitney test for two independent samples, categorical variables were analyzed with Cramer’s V test. The results indicated that participants with GJH exhibited inferior balance and back muscle static strength endurance but greater flexibility compared to those without GJH. Significant differences were observed in foot posture. However, handgrip strength, explosive strength, and abdominal muscle static strength endurance did not differ significantly between groups. No significant differences were observed in HR-QoL components between the two groups. In conclusion, there appears to be a link between GJH and increased flexibility, impaired balance, reduced back muscle static strength endurance, and altered posture of both feet.

## 1. Introduction

Generalized joint hypermobility (GJH) is a common condition that refers to greater than normal joint laxity and excessive range of motion (ROM) across multiple joints [1,2]. GJH is assessed via the Beighton score, which evaluates hypermobility at nine joints [3].

Since 2017, the classification of joint hypermobility has changed. Joint hypermobility syndrome is no longer the appropriate terminology for newly diagnosed patients. The hypermobility spectrum disorders (HSDs) were created, ranging from asymptomatic joint hypermobility to hypermobile EDS (hEDS). The Ehlers–Danlos syndromes (EDS) are currently classified into a system of thirteen subtypes. Generalized joint hypermobility (GJH), which belongs to the components of HDS, can be symptomatic or asymptomatic.

Although joint laxity, or hypermobility, is a well-known condition, there has long been a lack of a universally acknowledged definition or terminology [1]. An excessive range of motion across multiple joints is usually referred to as generalized joint hypermobility (GJH). The terms joint hypermobility syndrome and benign joint hypermobility syndrome (BJHS) were used to describe a disorder characterized by musculoskeletal symptoms, such as chronic joint or ligament pain or osteoarthritis, due to joint hypermobility [4,5,6].

In recent years, GJH and joint hypermobility syndrome (JHS) have started to gain attention as potential sources of pain and injury among college students [4,7]. Subsequently, a more precise stratification between individuals at high risk and those at no risk was warranted [1]. Individuals with hypermobility often seek physical therapy for acute and chronic musculoskeletal difficulties [8]. Within clinical settings, joint hypermobility is generally overlooked, with a prevalence of polyarticular hypermobility around 2–35% in males and 5–57% in females, depending on age and race [9]. Given the high prevalence of GJH among females, studies to unravel the underlying mechanism of the condition in females may be relevant. To that end, investigating the presence or absence of postural and musculoskeletal comorbidities such as idiopathic scoliosis, flat feet, impaired proprioception, joint instability, ligament injuries, and increased risk of reinjuries, joint dislocations, and/or poor health-related quality of life can present unique benefits [2,10,11,12,13,14].

Of specific interest herein, joint hypermobility affects overall posture [14]. Increased joint mobility can also be associated with altered foot posture. For instance, ligament laxity and whole-body hypermobility were linked to foot pronation [15]. A correlation is also apparent between hypermobility of the first ray and Hallux Valgus deformity [16] and planus foot type [17]. Finally, alterations in the overall body and foot alignment and their associations with musculoskeletal symptomology may also be observed in cases of joint laxity.

In general, the specifics of physical fitness in hypermobile individuals and the effects of increased joint flexibility on physical performance are still relatively unknown. Physical fitness is a construct of health encompassing muscular strength and endurance, flexibility, balance, coordination, and cardiovascular fitness [18,19]. Some scientific sources suggest that the handgrip strength [20] and jumping capacity [21] of hypermobile individuals may be reduced. GJH is often accompanied by muscle weakness and even atrophy of the muscles, which further exacerbates the stability of the joint [22]. Individuals with hypermobility may suffer from reduced muscle strength [23], decreased endurance, and decreased functional status [21]. The stability of the hypermobile joint may be decreased, which may cause muscle strain, muscle tension, muscle spasm, tendonitis, and/or pain. “The hypermobile joint often alters the biomechanics of the body, causing compensatory changes at other body sites and further strain” [22]. Thus, we find that it should be possible for people with joint hypermobility to focus on strengthening their muscles to prevent these symptoms, especially given that muscle-strengthening exercises reduce the risk of injury and have potential benefits for the musculoskeletal system [24]. Balance, another physical characteristic that is important for health, is occasionally presented as an issue among individuals with joint hypermobility [25,26]. These people may also have a greater fear of falling [26].

The scientific literature suggests that GJH individuals may have impaired health-related quality of life (HR-QoL) [22,27]. Briefly defined, HR-QoL refers to a synthesis of several health domains, including the physical, psychological, and social, all of which are affected by individual experiences, expectations, beliefs, and perceptions [28]. Consequently, a comprehensive understanding of GJH may require considering both its physiological and psychological components. We hypothesize that physical fitness characteristics and health-related quality of life (HR-QoL) will differ between young women with and without GJH. The purpose of this study was to identify characteristics of physical fitness and HR-QoL in college females with GJH.

## 2. Materials and Methods

### 2.1. Participants

This study was conducted over a span of six months, from May to October. The approval for this study was granted by the Bioethical Center of Lithuanian University of Health Sciences (approval No. BEC-SR(M)-224). The data collection for this study was carried out according to the Helsinki Declaration. Participation in this study was voluntary, and written informed consent was obtained prior to participation. Participants were recruited by means of an announcement of a research project at the university. A total of 67 (the mean age 20.08 SD 1.99) individuals met the inclusion criteria. In order to participate in this study, participants needed to be (a) female and (b) between 18 and 44 years of age [29]. Individuals participating in professional sports such as flexibility training (e.g., ballet, gymnastics), meeting the WHO physical activity recommendations [29], and presenting comorbidities (e.g., diabetes mellitus, hypertension, liver cirrhosis, heart failure) could not participate in this study.

### 2.2. GJH Assessment

**Figure 1:**
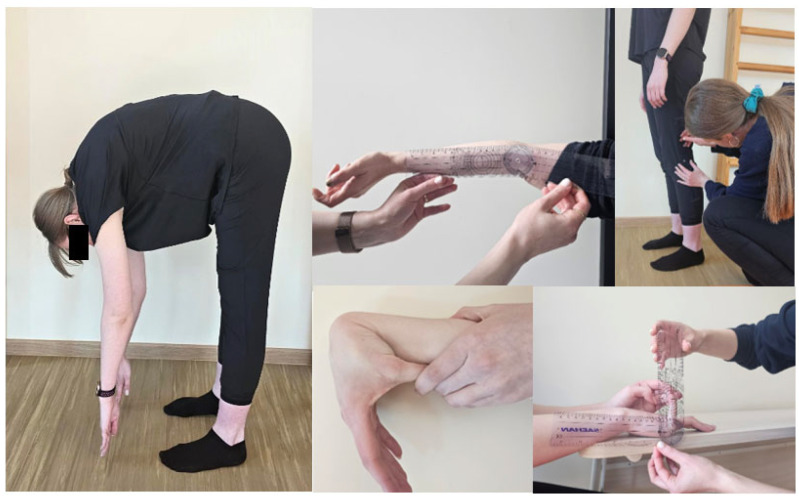
Beighton scale assessment.

Hypermobility was assessed using the 9-point Beighton score [30]. The ability to perform each of the following tasks was evaluated: (1) forward flexion of the trunk with knees straight, palms resting comfortably on the floor; (2) >10° of passive elbow hyperextension; (3) >10° of passive knee hyperextension; (4) passive opposing of the thumb to the flexor of the forearm; and (5) passive dorsiflexion of the hand’s fifth digit >90°. Items 2 to 5 were examined bilaterally, allocating one point per side (see Figure 1). The goniometer was used to measure the passive bilateral dorsiflexion of the fifth metacarpophalangeal joint and the passive bilateral hyperextension of the elbow and knee. The goniometer (SAEHAN^®^, South Korea) is a plastic instrument with two arms, one fixed and one mobile. It is marked with scales that allow the measurement of joint range angles. A measurement sensitivity is 2 degrees [31].

**Figure 2:**
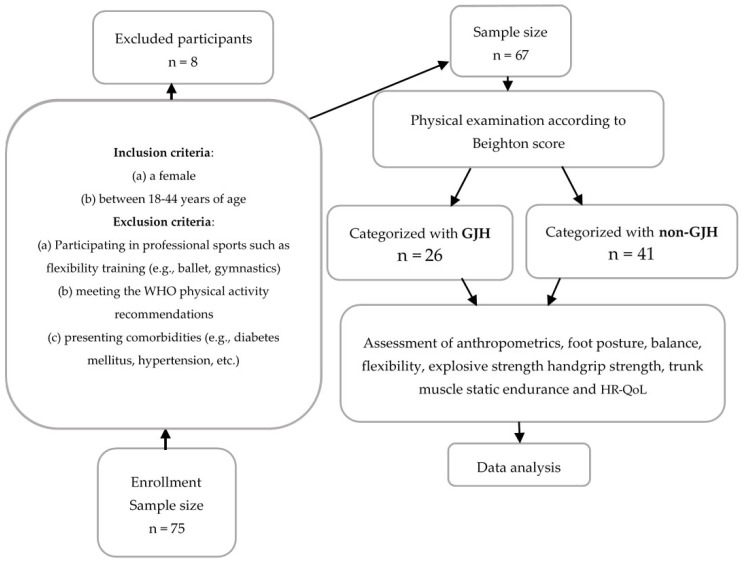
Flowchart of participants.

Participants‘ total scores ranged from 0 to 9. A score of ≥5 was used as the standard cutoff score to indicate the presence of GJH [32]. The test has moderate-to-high inter-tester repeatability [33] and has demonstrated validity and reliability in a number of studies [5,6,34,35,36]. According to their Beighton score, which assesses hypermobility, participants were divided into two groups: those with GJH (Beighton score ≥ 5) and those without GJH (Beighton score < 5) [37,38] (see Figure 2).

### 2.3. Anthropometric Measurement

Height (in centimeters) was measured to the nearest 0.1 cm by means of a medical stadiometer (Charder HM200M, Charder Scales, Taichung City, Taiwan). Weight was measured via a digital Tanita Scale plus BIA (Model BF-545, Tanita; Arlington Heights, IL, USA) weighing scale with 0.1 kg sensitivity. Participants were instructed to remove their shoes and heavy outfits prior to measurements. Body mass index (BMI) was calculated using Quetelet‘s equation (BMI = weight (kg)/height (m)^2^) [39]. In order to ensure the predictive accuracy of these equations, participants were instructed to follow the Tanita Scale plus BIA testing guidelines [40].

### 2.4. Foot Posture Analysis

Foot posture index-6 (FPI-6) was used to analyze foot posture [41]. FPI-6 classifies foot posture based on six items. Prior to administering FPI-6, participants were instructed to stand in a relaxed position with both feet firmly grounded on the floor. Next, palpation and observation of the following foot regions took place: (1) palpation of the talus head position, observation of (2) curvatures above and below the lateral malleolus, (3) position of the calcaneus in the frontal plane, (4) prominence in the talonavicular joint, (5) the medial longitudinal arch’s congruence, and (6) abduction/adduction of the forefoot. Each criterion was scored in [−2 to 2] range, resulting in a total score of −12 to 12 points. Negative values indicated supinated foot posture. Positive values indicated pronated foot posture. A score of 0 was given for normal or neutral foot positions [42]. The normal range of the FPI-6 was 0 to +5, FPI-6 values above and below this rank were considered abnormal feet [43].

### 2.5. Assessment of Physical Fitness

We assessed the components of physical fitness, such as balance, flexibility, and muscle strength, because they are the most health-related.

#### 2.5.1. Balance Assessment

For the assessment of the static balance, participants performed a flamingo balance test [44]. A standard balance board with a length of 50 cm, a height of 4 cm, and a width of 3 cm was used for the flamingo balance test. Participants were instructed to stand barefoot on the balance board with their preferred foot, bend their free leg at the knee, and hold this close to their buttocks. The foot of that leg was held with the hand of the same side. The chronometer was started when participants stated that they were ready. If participants lost their balance (i.e., touched the floor, fell, or stopped holding the foot), the time was stopped. Then participants had to climb on the balance board again as quickly as possible and resume the instructed position. Once the position was stabilized, the time count was continued. The total number of attempts to keep the target position for 60 s was recorded. If participants fell more than 15 times in the first 30 s, the test was terminated. A lower number of attempts (climbs on the balance board) in the flamingo balance test indicated higher balance ability [44,45].

#### 2.5.2. Flexibility

Flexibility was measured using the sit and reach test. Participants sat on the floor with straight legs. The feet were placed against the standard test box, and the shoes were removed. Participants were instructed to lean as forward as possible along the measuring line. The ruler had to be pushed with the fingertips in a smooth and slow movement. The best of two attempts was recorded. Better flexibility was indicated with longer-reach distances [44].

#### 2.5.3. Explosive Strength Assessment

Explosive strength was measured by a standing broad jump test, using a tape measure on the floor. Participants were instructed to stand behind a line marked on the ground with feet slightly apart and jump forward as far as possible, using arm swings and knee bending before jumping. The distance jumped was recorded from the take offline to the nearest point of contact on the landing (back of the heels). The best of two attempts was recorded. Higher distances (in centimeters) indicated greater explosive strength [44].

#### 2.5.4. Handgrip Strength Assessment

Handgrip strength was assessed using a hydraulic hand dynamometer (SAEHAN^®^ grip dynamometer, Model SH5001; SAEHAN Corporation, YangdeokDong, Masan, South Korea). The test was performed in a standing position, holding a dynamometer in the preferred arm with a flexed forearm at 90 degrees and the palm facing inwards. Participants were instructed to squeeze the dynamometer as forcefully as possible [44]. The best of two attempts was recorded. Higher squeezed kilograms indicated stronger handgrip strength [46].

#### 2.5.5. Trunk Muscle Static Strength Endurance Assessment

To assess back muscle static strength endurance, participants were instructed to lie down on the mat in a prone position with their hands by their sides [47]. After contracting the muscles of the gluteal region, participants were instructed to “raise the head and chest from the mat (not touching the mat), avoiding over extension and holding the position for 5 min or until presence pain or major discomfort”. For the abdominal muscle static strength endurance assessment, participants lay down on the mat in a supine position with their feet pelvis width apart and knees flexed (90 degrees). Participants were instructed to “bend forward with extended hands, reaching knee joints but not holding onto them, and stay in this position for 5 min or until the presence of pain or major discomfort”. Longer time spent in each of the instructed positions indicated either better back or abdomen muscle static strength endurance.

### 2.6. Health-Related Quality of Life

HR-QoL was evaluated through the SF-36 Quality of life questionnaire. The SF-36 includes 36 questions that are classified into 8 scales that assess participants perceived quality in the following health-related domains: physical functioning, physical limitation, bodily pain, general health, vitality (energy and fatigue), social functioning, emotional limitation, and mental health. The raw scores of each domain were converted into a 0–100 scale, with higher scores indicating a higher HR-QoL [48].

### 2.7. Data Analysis

Data analysis was computed using IBM SPSS 27.0 software (New York, NY, USA, released on 19 June 2020). For descriptive statistics, median, interquartile range (first and third quartiles), mean, standard deviation (SD), and percentage values were calculated. For the comparison of two independent samples, the Mann–Whitney test was used. Categorical variables were analyzed with Cramer’s V test. Statistical significance was set at *p* < 0.05.

## 3. Results

### 3.1. Participants’ Data

Of all participants, 38.8% were found to present with GJH (see Table 1). There were no significant differences between participants with GJH and without GJH based on age, height, weight, or BMI.

### 3.2. Foot Posture

The results of the left foot posture index revealed a significant difference between participants with and without GJH (Cramer’s V 1; *p* < 0.001). Specifically, 61.5% of participants with GJH and 31.7% of participants without GJH exhibited an altered posture in their left foot. Similarly, for the right foot, significant differences were observed between the groups (Cramer’s V 0.975; *p* < 0.001). Most participants with GJH (73.1%) demonstrated alterations (tendency to pronate feet) in the right foot, while only 39% of participants without GJH exhibited altered right foot posture.

### 3.3. Physical Fitness

There was a significant difference between participants with GJH and participants without GJH in flexibility (sit and reach test) (U = 252; Z = −3.62; *p* < 0.001); as expected, flexibility was better among GJH individuals and static balance (flamingo balance test) (U = 334.5; Z = −2.562; *p* = 0.01), which means that GJH participants had worse balance (see Table 2).

Participants with GJH and participants without GJH had similar handgrip strength (U = 459; Z = −0.955; *p* = 0.34). Additionally, comparable explosive strength distances emerged for the lower extremities in both groups (U = 397.5; Z = −1.74; *p* = 0.08). However, participants without GJH achieved better results in the back muscle static strength endurance test compared to participants with GJH (U = 368; Z = −2.2; *p* = 0.027). No statistically significant difference in abdomen muscle static strength endurance emerged between groups (U = 513.5; Z = −0.26; *p* = 0.79).

### 3.4. Health-Related Quality of Life

Components of HR-QoL did not differ between groups (Table 3).

## 4. Discussion

We hypothesize that physical fitness characteristics and health-related quality of life (HR-QoL) will differ between young women with and without GJH. The purpose of this study was to identify the characteristics of physical fitness and HR-QoL in college females with GJH. This study partially supports the hypothesis that there are differences in physical fitness characteristics and health-related quality of life (HR-QoL) between young women with and without generalized joint hypermobility (GJH). In this study, physical fitness variables such as balance, flexibility, back muscle strength endurance, and foot posture showed important differences between the GJH and non-GJH groups. On the other hand, we found no significant differences in the components of health-related quality of life (HR-QoL) between both groups.

In this study, we used a complex assessment of GJH individuals and analyzed for differences with non-GJH individuals and associative relationships. We believe that the existing knowledge will help physiotherapists, sports medicine and rehabilitation physicians, and public health professionals better understand aspects of physical performance and health-related quality of life (HR-QoL) in individuals with joint hypermobility. This knowledge can be integrated into clinical practice, to assess the physical fitness and foot posture of patients more closely with GJH and facilitate the design of rehabilitation or prevention programs based on these findings.

### 4.1. Physical Fitness

In our cohort, individuals with and without GJH were found to have similar handgrip strength and explosive strength. Other authors have also indicated that muscle strength and explosive strength can be the same in individuals with and without hypermobility [49,50]. For instance, increased ROM of joints of the upper extremity does not influence handgrip strength [51]. This said, in contrast to our findings, early evidence has suggested that GJH can be associated with decreased muscle strength for all muscle groups [21].

In our study, individuals with GJH showed lower back muscle static strength endurance compared with the non-GJH group. No scientific data could be found comparing static strength endurance of trunk muscles (abdominal and back) between GJH and non-GJH subjects; however, there is scientific evidence that persons with joint hypermobility have partially impaired lateral abdominal muscle function given a lesser ability to increase transversal abdominal muscle thickness during contraction compared with those without joint hypermobility [52].

In addition, balance appeared to be worse for individuals with GJH than those without GJH. Previous work has also shown impairments of balance in adults with GJH [53,54]. This may help explain the results of the flamingo balance test in the present study. Specifically, present participants with joint hypermobility may have impaired proprioception [12,55,56] that may have affected their attempts to maintain balance.

### 4.2. Foot Posture

The results of our study showed significant differences in both feet between the GJH and non-GJH groups. The majority of GJH participants had altered their left and right feet. Other scientific sources state that there is a relationship between ligament laxity and foot pronation [15,57]. In contrast, there is a study suggesting that joint hypermobility does not affect the medial longitudinal arches of the feet in adults [49]. And here is another study even claiming that 45% of adults with joint hypermobility syndrome had a high arch of the longitudinal arch of the feet, 27.5% of people were within the normal range, and 27.5% presented a lower arch [58]. Increased midfoot loading was found in female athletes with GJH [59].

### 4.3. Health-Related Quality of Life

Our results suggest that individuals with GJH have the same HR-QoL as those without GJH. To support the present findings, previous work has found no significant differences with regards to HR-QoL between those with and without GJH [60]. On the other hand, there is scientific evidence that joint hypermobility is associated with worse quality of life [61]; research shows that physical and mental component scores of SF-36, as well as all subgroup scores except social function, were significantly lower in hypermobile participants.

### 4.4. Strengths and Limitations of the Study

This study had several strengths. In the present study, participants were examined by a physical therapist with at least 10 years of experience. The results from this study may help therapists assess, evaluate, and correct possible balance impairments in individuals with joint hypermobility. Educating individuals with joint hypermobility about their specific balance impairments and providing strategies for improvement empowers them to take an active role in managing their condition. This education can lead to better self-management and a decreased reliance on healthcare services over time. Also, based on the present results, considering that individuals with hypermobility may have an imbalance in trunk muscle static strength endurance, worse balance, and foot posture, therapists may benefit from developing health or rehabilitation programs targeting trunk muscle static strength endurance, balance, and foot posture in individuals with hypermobility. We firmly believe that it is important to know the differences in physical fitness between people with GJH and without GJH due to the correct selection of physical exercises and rehabilitation to form guidelines on how to safely train and rehabilitate people with GJH.

Nevertheless, the present study included several limitations. First, arm and leg dominance in the present study was unknown to researchers. This information could have helped to further refine the results associated with foot posture or handgrip strength. Second, in this study, we did not collect data related to lifestyle habits (i.e., food choices, harmful habits, sleep quality, etc.). This data could have helped interpret the results of muscle testing and scores associated with HR-QoL better.

### 4.5. Future Studies

There are many inconsistencies in the scientific literature on the prevalence of GJH [2,9,21]. It would be useful to clarify the prevalence of GJH among young and middle-aged individuals. It would be beneficial to perform a more comprehensive and integrated assessment of the musculoskeletal system in GJH subjects, including measurement of body posture in all planes, not just foot posture. To determine whether GJH individuals differ from the non-GJH population in any of the musculoskeletal and postural characteristics. Upcoming studies may adapt the Bristol impact of the hypermobility questionnaire [62] along with objective measures of the musculoskeletal system to achieve more accurate physical fitness assessments. This is important because once the presence of GJH is detected, results from these assessments could facilitate highly targeted therapy prescriptions. These prescriptions could guide individuals at risk to manage their condition and prevent potential decline. Finally, upcoming research could investigate training effects on hypermobility, and test the effectiveness of alternative strategies to manage hypermobility and its associated conditions in diverse populations.

## 5. Conclusions

The evident association between GJH and heightened flexibility, impaired balance, reduced back muscle static strength endurance, and altered foot posture highlights the profound impact of this condition on musculoskeletal health and functional capabilities. Understanding the correlation between these physical characteristics and health-related quality of life is paramount, given the substantial influence of GJH on daily functioning and overall well-being in affected individuals. Moreover, our findings underscore the importance of tailored assessment and intervention strategies for individuals with GJH to address specific physical challenges and improve overall musculoskeletal health and functional abilities.

## Figures and Tables

**Table 1:** Characteristics of participants with GJH and from the non-GJH.

	GJH		Non-GJH		
	n = 26		n = 41		
	5.29 (5–7)		1.8 (0–4)		
	Mean (SD)	Me (IQR)	Mean (SD)	Me (IQR)	U, Z, *p* Value
Age (years)	20.06 (1.2)	20 (19–21)	20.15 (2.2)	19.5 (19–21)	462.5; −0.949; 0.343
Height (cm)	168.5 (5.2)	168 (165–172)	168.4 (6.7)	169 (165–173)	500; −0.425; 0.671
Weight (kg)	59.81 (6.16)	59.6 (55.2–63.25)	60.96 (8.76)	59.3 (56.05–64.2)	516; −0.219; 0.827
BMI (kg/m^2^)	21.1 (1.8)	20.55 (19.45–22.92)	21.5 (2.39)	21 (19.57–22.15)	520; −0.167; 0.867

Note: GJH: generalized joint hypermobility; IQR: interquartile range; and SD: standard deviation.

**Table 2:** The comparison of flexibility, balance, strength, power, and muscle static strength endurance between GJH and non-GJH.

	GJH		Non-GJH			
	n = 26		n = 41			
	Mean (SD)	Me (IQR)	Mean (SD)	Me (IQR)	U, Z, *p* Value	r
Sit and reach test (cm)	33.28 (5.05)	32.7 (30–37.5)	26.5 (7.56)	28 (21–31.5)	**252; −3.62; <0.001**	0.442
Flamingo balance test (attempts)	9.73 (5.2)	9 (6–12)	6.6 (4.4)	6 (3–9)	**334.5; −2.562; 0.01**	0.313
Handgrip strength test (kg)	29.95 (4.76)	29.5 (26–33)	30.85 (5.82)	31 (26–35.5)	459; −0.955; 0.34	0.117
Standing broad jump test (m)	1.56 (0.24)	1.61 (1.35–1.69)	1.64 (0.2)	1.7 (1.56–1.77)	397.5; −1.744; 0.081	0.213
Abdomen muscle static strength endurance test (s)	197.6 (103)	210 (82–300)	201 (104.74)	240 (82–300)	513.5; −0.262; 0.793	0.032
Back muscle static strength endurance test (s)	208.65 (70.7)	192.5 (166–300)	242.66 (76.75)	300 (205–300)	**368; −2.206; 0.027**	0.269

Notes: GJH: generalized joint hypermobility; Me: median: IQR: interquartile range; SD: standard deviation; and r: effect size. Bold *p* values indicate statistical significance.

**Table 3:** The comparison of SF-36 questionnaire‘s (which is designed to assess HR-QoL) domain scores in GJH and non-GJH.

	GJH		Non-GJH			
	n = 26		n = 41			
	Mean (SD)	Me (IQR)	Mean (SD)	Me (IQR)	U, Z, *p* Value	r
Physical functioning	95 (6.48)	95 (90–100)	95.6 (6.34)	95 (95–100)	483; −0.693; 0.488	0.084
Physical limitation	92 (17.26)	100 (87.5–100)	92.1 (14.19)	100 (75–100)	513.5; −0.342; 0.741	0.04
Bodily pain	85.54 (19.08)	89 (78–100)	82.54 (18.66)	89 (72.5–100)	462; −0.951; 0.342	0.116
General health	76.6 (16.38)	75 (67.5–75)	72.2 (14.71)	75 (60–85)	456.5; −0.991; 0.321	0.121
Vitality	62.31 (17.16)	65 (53.75–76.25)	66.7 (12.74)	65 (57.5–77.5)	478.5; −0.706; 0.48	0.086
Social functioning	85.92 (22.18)	94.5 (78–100)	89.6 (10.1)	89 (78–100)	508; −0.342; 0.732	0.042
Emotional limitation	88.48 (23.21)	100 (94.5–100)	90 (21.4)	100 (100–100)	418; −1.495; 0.135	0.182
Mental health	74.4 (19.46)	80 (64–82)	70.15 (14.29)	76 (60–80)	504; −0.15; 0.88	0.018

Note: GJH: generalized joint hypermobility; Me: median; IQR: interquartile range; SD: standard deviation; r: effect size.

## Data Availability

The data used and analyzed during the current study are available at figshare.com, https://doi.org/10.6084/m9.figshare.25615293.
